# Human B-1 Cells and B-1 Cell Antibodies Change With Advancing Age

**DOI:** 10.3389/fimmu.2019.00483

**Published:** 2019-03-19

**Authors:** Nely Rodriguez-Zhurbenko, Tam D. Quach, Thomas J. Hopkins, Thomas L. Rothstein, Ana M. Hernandez

**Affiliations:** ^1^Center of Molecular Immunology, Havana, Cuba; ^2^Center for Autoimmune Musculoskeletal and Hematopoietic Diseases, The Feinstein Institute for Medical Research, Manhasset, NY, United States; ^3^Center for Oncology and Cell Biology, The Feinstein Institute for Medical Research, Manhasset, NY, United States; ^4^Center for Immunobiology and Department of Biomedical Sciences, Western Michigan University Homer Stryker MD School of Medicine, Kalamazoo, MI, United States

**Keywords:** human B-1 cells, aging, IgM, antibody secretion, repertoire

## Abstract

Age-related deficits in the immune system have been associated with an increased incidence of infections, autoimmune diseases, and cancer. Human B cell populations change quantitatively and qualitatively in the elderly. However, the function of human B-1 cells, which play critical anti-microbial and housekeeping roles, have not been studied in the older age population. In the present work, we analyzed how the frequency, function and repertoire of human peripheral blood B-1 cells (CD19+CD20+CD27+CD38^low/int^CD43+) change with age. Our results show that not only the percentage of B-1 cells but also their ability to spontaneously secrete IgM decreased with age. Further, expression levels of the transcription factors XBP-1 and Blimp-1 were significantly lower, while PAX-5, characteristic of non-secreting B cells, was significantly higher, in healthy donors over 65 years (old) as compared to healthy donors between 20 and 45 years (young). To further characterize the B-1 cell population in older individuals, we performed single cell sequencing analysis of IgM heavy chains from healthy young and old donors. We found reduced repertoire diversity of IgM antibodies in B-1 cells from older donors as well as differences in usage of certain VH and DH specific genes, as compared to younger. Overall, our results show impairment of the human B-1 cell population with advancing age, which might impact the quality of life and onset of disease within the elderly population.

## Introduction

Life expectancy is rapidly increasing worldwide. However, age-related diseases such as atherosclerosis, cancer, autoimmunity, type-2 diabetes mellitus, and microbial infection still have an impact on morbidity and mortality of elderly adults. Further, aging is accompanied by broad structural and functional changes in the immune system, usually related to increased susceptibility to the aforementioned diseases (1) and to decreased response to vaccination (2, 3).

It has been demonstrated that advancing age exerts a strong influence on remodeling of the B cell compartment of the immune system [reviewed in (4)]. There are two primary branches within the B cell population: conventional B-2 cells and B-1 cells. In mice, B-1 and B-2 cells differ in function and development, with B-2 cells originating mainly from the bone marrow and B-1 cells mainly from fetal liver and to a lesser extent from adult bone marrow (5, 6). Conventional B-2 cells cooperate with T cells in the germinal center to provide high-affinity long lasting antibody responses (7). On the other hand, B-1 cells spontaneously secrete natural antibodies mainly against non-protein antigens in the absence of exogenous immunization. This feature allows B-1 cells to provide pre-existing, immediate defense to counteract microbial infection (8–10). Given that B-1 cells are among the first B cells to develop, and the fact that B-1 cells, particularly IgM-secreting B-1 cells, are already present before birth, B-1 cells may be considered as the principal B cells responsible for establishing a natural antibody repertoire and defense early in ontogeny. Furthermore, it has been suggested that the earliest waves of B-1 cells during ontogeny produce natural IgM that regulates both B-1 and B-2 cell development (11). Also, B-1 cell antibodies influence some chronic diseases, such as atherosclerosis [reviewed in (12)].

Unlike high-affinity antibodies produced by B-2 cells, antibodies secreted by B-1 cells in mice have a germ-line like structure, due to minimal insertion of non-template-encoded nucleotides (N-region addition) and little somatic hypermutation (13, 14). B-1 cell-derived antibodies are often autoreactive, and serve a homeostatic function in speeding disposal of apoptotic cell debris and noxious molecular species [reviewed in (15)]. As such, B-1 cell antibodies can protect against atherosclerosis and autoimmunity (16, 17). Further, B-1 cells are considered more effective antigen presenting cells (APCs) than B-2 cells, and B-1 cells, but not B-2 cells, stimulate CD4+ T cells to differentiate into Th17 effector cells (18).

In humans, B-1 cells have been identified as CD20+CD27+CD38^low/int^CD43+ based on the fact that B cells with this phenotype fulfill key functional criteria characteristic of mouse B-1 cells, such as spontaneous antibody secretion (19, 20). This population has been evaluated in healthy donors and in patients with autoimmune diseases, suggesting a role for human B-1 cells in such situations (21). For example, the frequency of B-1 cells in patients with relapsing-remitting multiple sclerosis was decreased compared with healthy controls (22). Also, the human B cell population with the phenotype CD20+CD27+CD43+CD5- generates antibodies to capsular polysaccharides of *Streptococcus pneumoniae* (23) suggesting an important role of this population in fighting infection.

Several reports have shown changes in conventional B-2 cells during aging, both in mice and humans. There is a decline in total B cell number or frequency during aging, which is more clearcut in humans than in mice (4). Further, the proportion of different subtypes within the B-cell lineage changes with age. For example, marginal zone (MZ) B cells significantly decline in aged BALB/c mice (24) while there is an increase in age-associated B cells (ABCs) (25). This is more controversial in the human scenario: different subsets of B cells have been shown to increase or decrease during aging depending on the cell phenotype or age of the cohort (26, 27). Functionally, aging impacts the mature B cell antibody response to vaccination. After antigenic challenge, B cells from old individuals produce fewer antibodies (28) and are impaired in the ability to undergo class switch recombination (CSR) (29, 30) and somatic hypermutation (SHM) (31), as compared to young individuals. This is compounded by loss of diversity in the B cell repertoire (32). As a result, antibodies generated in both old mice and old humans are less protective compared with antibodies produced by young adults (33, 34).

On the other hand, the impact of aging on the frequency and function of B-1 cells has been less studied. The most noted feature of B-1 cells in the aging mouse immune system is a change in repertoire. For example, certain VH11-encoded PtC-binding IgH sequences increase progressively with age in the pre-immune B-1a IgH repertoire (35). Other important specificities of B-1 cells are phosphorylcholine (PC) (36) and pneumococcal capsular polysaccharides, antigens found on the cell walls of the bacteria *Streptococcus pneumoniae* (10, 37). These bacteria are responsible for pneumococcal infections which are dramatically increased in old relative to young adults (38). The importance of B-1a cells in protection against pneumococci is indicated by experiments showing that in the absence of B-1a cells animals were unable to survive infection because of the lack of natural IgM, especially anti-PC and anti-pneumococcal capsular polysaccharide (PPS)-3 (10). Natural anti-pneumococcal antibodies produced by B-1 cells are increasingly important in aging since in the old population the adaptive anti-pneumococcal antibody response generated after immunization is less protective both in mice and humans (38–42). However, it has been shown that protection afforded by natural serum IgM against pneumococcal infection in mice diminishes with advancing age (43). B-1 cells isolated from old mice produce antibodies with more N-addition than B-1 cells from young animals (43–45), which can contribute to the above mentioned impaired protection. Still, a comprehensive study of changes in human B-1 cells during normal aging is lacking.

In the current work we elucidated age-related changes in the human B-1 cell population. We found B-1 cell frequency significantly decreases with advancing age. Functional analysis showed the B-1 cell population from older donors contains fewer antibody secreting cells than does the B-1 cell population present in younger donors. Further, we found transcription factors involved in the “classical” immunoglobulin secretion pathway are differentially expressed in B-1 cells from young and old donors. Importantly, we found reduced IgM repertoire diversity in B-1 cells from older as compared to younger donors. However, there were no differences in B-1 cell IgM somatic hypermutation or N-addition between young and old donors. Thus, with advancing age there are fewer B-1 cells, a smaller fraction of which secrete IgM, that display diminished diversity. Overall, these data indicate a decline in B-1 cell functionality in older individuals that might compromise the ability to oppose age-related diseases such as microbial infection and atherosclerosis.

## Materials and Methods

### Human Samples

Adult peripheral blood samples were obtained by venipuncture from a total of 120 healthy donors (20–88 years), of which 58% were females and 42% males. Donors were selected on the basis of good health status and no reports of infection/immunization within 4 weeks of blood draws. This study was conducted in accordance with the World Medical Association's Declaration of Helsinki. Written informed consent was received from all donors. This study was approved by the North Shore-LIJ Health System Institutional Review Board.

### Cell Isolation

Heparinized blood samples were processed immediately upon receipt. Human peripheral blood mononuclear cells (PBMCs) were isolated by density gradient centrifugation using lymphocyte separation medium (Cellgro). Mononuclear cells were washed in phosphate-buffered saline (PBS) containing 1 mM EDTA and resuspended in either cell-sorting buffer [0.5% bovine serum albumin (BSA) in PBS] or FACS buffer [2.5% fetal calf serum (FCS), 1 mM EDTA, 0.02% NaN3 in PBS].

### Cell Sorting

PBMCs were treated with 5% normal mouse serum (NMS), stained with an antibody mixture consisting of anti–CD19-allophycocyanin-Alexa Fluor700, anti–CD27-allophycocyanin, anti–CD43-FITC, anti–CD38-PerCP-Cy5.5, anti–CD20-Pacific Blue, anti–CD3-PE-TexasRed, anti–CD4-PE-TexasRed, and anti–CD7-PE-TexasRed in cell-sorting buffer, and then stained with Aqua Live/Dead dye (Invitrogen). Fluorescence Minus One controls were used for CD43+ and CD27+ cell selection. After washing, cells were resuspended in cell-sorting buffer and sort-purified on an Influx instrument (BD). B-1 (CD19+CD20+CD27+CD38^low/int^CD43+) cells were collected from the CD3/4/7 negative population as previously described (20), following the gating strategy represented in Supplementary Figure 1A. The average purity of purified B-1 cells post-sort was 85%. For single-cell antibody sequencing, purified B-1 cells were re-sorted immediately after the initial sort, according to CD19+CD20+CD27+CD38^low/int^CD43+ expression applying FSC-H/FSC-W-based doublet discrimination and single sort mask settings (Supplementary Figure 1B).

### Flow Cytometry

Single-cell suspensions of PBMCs were pre-incubated with NMS for 20 min and then labeled for 30 min at 4°C with the following antibodies in FACS buffer: anti–CD19-Alexa Fluor 700, anti–CD20-Pacific Blue, anti–CD3-PE-TexasRed, anti–CD4-PE-TexasRed, anti–CD7-PE-TexasRed, anti–CD27-allophycocyanin, anti–CD38-PerCP-Cy5.5, anti–CD43-allophycocyanin-Alexa Fluor 750, and Aqua Live/Dead dye. Expression of cell surface markers was analyzed with a Gallios flow cytometer and FlowJo Software v. 9.7.6 (Tree Star). Antibodies were purchased from Beckman Coulter, BD Biosciences, or Biolegend.

### Enzyme-Linked Immunospot Assay (ELISPOT)

ELISPOT assays were used to detect antibody-secreting cells. Sort-purified cells (1–10 x 10^3^) were distributed on Multi-Screen-IP Plates (Millipore) precoated with goat anti–human IgM or goat anti–human IgG and then incubated in complete medium (RPMI medium supplemented with 10% FCS, 10 mM HEPES, 1 mM non-essential aminoacids, 2 mM L-glutamine, 1 mM pyruvate, and 50 μg/mL Penicillin/Streptomycin) for 18 h at 37°C and 5% CO_2_. Plates were then incubated with alkaline phosphatase-conjugated goat anti-human IgM or anti-human IgG (Southern Biotech), and developed with VECTOR Blue, Alkaline Phosphatase Substrate Kit III (Vector Laboratories). Immunoglobulin-secreting B cells were enumerated using C.T.L ImmunoSpot software (Cellular Technology Limited).

### Wright-Giemsa Staining

B-1 cell morphology was analyzed immediately after sorting by cytospin and Wright-Giemsa staining (Sigma-Aldrich) according to the manufacturer's protocol. Images were acquired on a Nikon E80i microscope or a Zeiss LSM 710. ImageJ/Fiji software was used for analysis.

### Real Time PCR

Total RNA from sort-purified B-1 cells was isolated using RNeasy (QIAGEN). cDNA synthesis was achieved using *i*Script (BioRad) according to the manufacturer's protocol. Quantitative PCR reactions were conducted in an ABI 7,300 machine using reagents and primers (Taqman) from Life Technologies. Primers: GAPDH Hs02758991_g1, Blimp-1 (PRDM-1) Hs00153357_m1, XBP-1 Hs0231936_m1, and PAX-5 Hs00277134_m1.

### Single-Cell RT-PCR and IGHM Analysis

Single cells were sorted into 96-well PCR plates containing 20 uL/well of ice-cold lysing buffer (40 U/μl RNaseOut, 0.1 M DTT, 0.4% IGEPAL, 1 mg/ml carrier RNA, and 1xRTIII buffer in dH_2_O), as previously reported (20). cDNA was synthesized in the original sorting plate in a final volume of 22.75 μl using SuperScript III RT (200 U/μl), random hexamers (50 μM), and dNTP mix (10 mM). Reverse transcription (RT) was performed at 42°C for 10 min, 25°C for 10 min, 50°C for 60 min, and 94°C for 5 min as described by Tiller et al. (46). IgH gene transcripts were amplified by two rounds of nested PCR. Protocol and primers were adapted from Tiller et al. (46). Two or more sequences were considered part of a clonotype if there was 100% homology in the CDR-H3 aminoacid sequence. The sequencing data have been submitted to the National Center for Biotechnology Information's Genbank (http://www.ncbi.nlm.nih.gov/genbank/) under accession numbers MK433645–MK434149.

### Statistics

Statistical analysis was carried out with GraphPad Prism 6.0 and SPSS 16.0 software. To examine differences between two or more groups, Mann-Whitney U-test and Dunn's test were used, respectively. To examine the relationships between two variables, Spearman's correlation analysis was performed. *P*-values for IgH gene repertoire analysis were calculated by Fisher's Exact Test and Chi-square test. Error bars represent standard error of the mean (SEM) unless otherwise indicated.

## Results

### Human B-1 Cells Decline With Advancing Age

**Figure 1:**
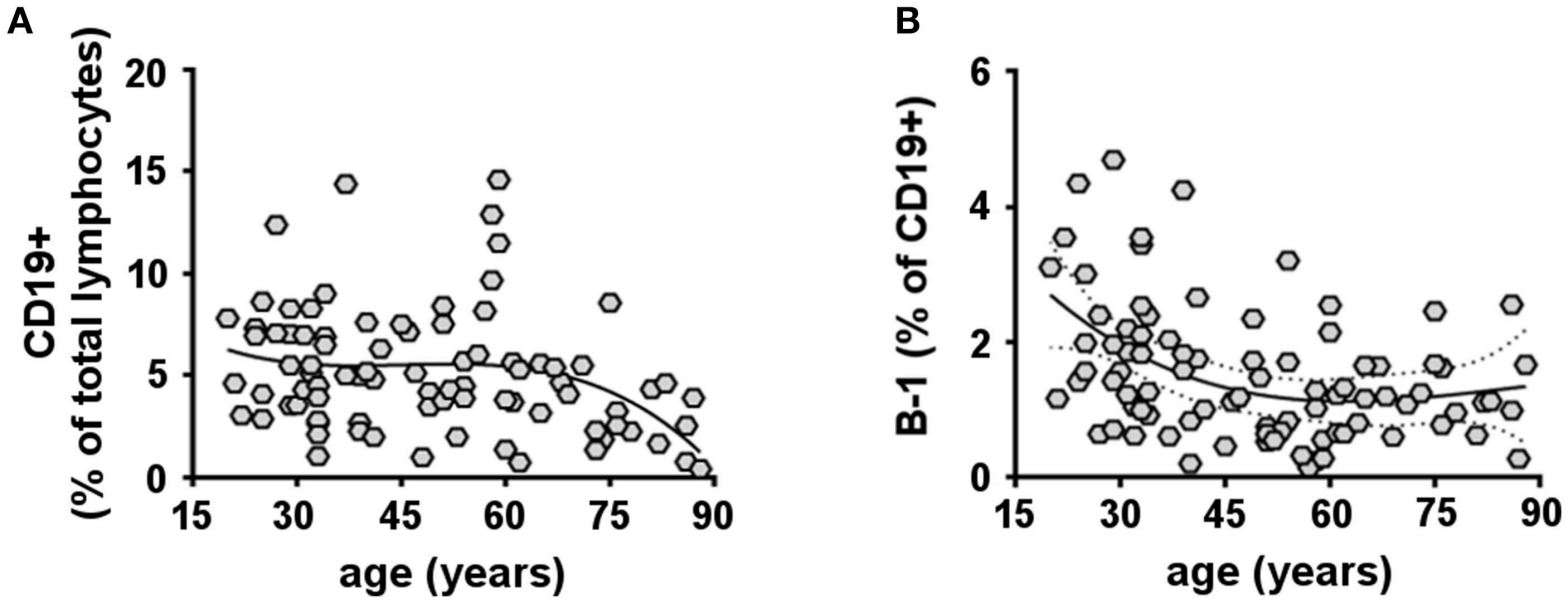
Human B-1 cells decline with advancing age. PBMCs isolated from 87 healthy donors (20–88 years) were analyzed by flow cytometry for total CD19+ B cells **(A)** or B-1 cells (CD19+CD20+CD27+CD38^low/int^CD43+) **(B)**. **(A**,**B)** Data plotted in each diagram represent CD19+ B cells given as percent lymphocytes **(A)** and B-1 cells given as percent CD19+ B cells **(B)**, each as a function of donor age. Spearman's r for each correlation and significance are the following: CD19+ B cells: *r* = −0.2997, *p* = 0.0053; B-1 cells: *r* = −0.3351, *p* = 0.0015. The solid line through the data points refers to a cubic regression curve fit.

The proportion of naïve and memory subsets within the human B cell compartment has been extensively studied [Reviewed in (47)]. However, data on age-related changes in the human B-1 cell (CD19+CD20+CD27+CD38^low/int^CD43+) population remain scarce. Here, we analyzed total CD19+ B cells and B-1 cells in the peripheral blood of 87 healthy donors of different ages (20–88 years). We found that the percentage of total CD19+ B cells as a fraction of total lymphocytes significantly decreased with advancing age (Figure 1A and Supplementary Figure 2A), consistent with previous reports (48–52). Importantly, we found the fraction of B-1 cells in the total CD19+ B cell population significantly declined with increasing age (Figure 1B). After analyzing various regression models, we found our data do not follow a linear regression trend and instead are better fit by a cubic model (higher R-squared). B-1 cell percentages were also compared grouping samples from donors within different age ranges. This analysis confirms a decrease in B-1 cell frequency with aging, which starts to be significant after the age of 50 (Supplementary Figure 2B). Thus, with advancing age, B-1 cells decline as a fraction of the declining total B cell population.

### Human B-1 Cell Spontaneous Antibody Secretion Declines With Advancing Age

**Figure 2:**
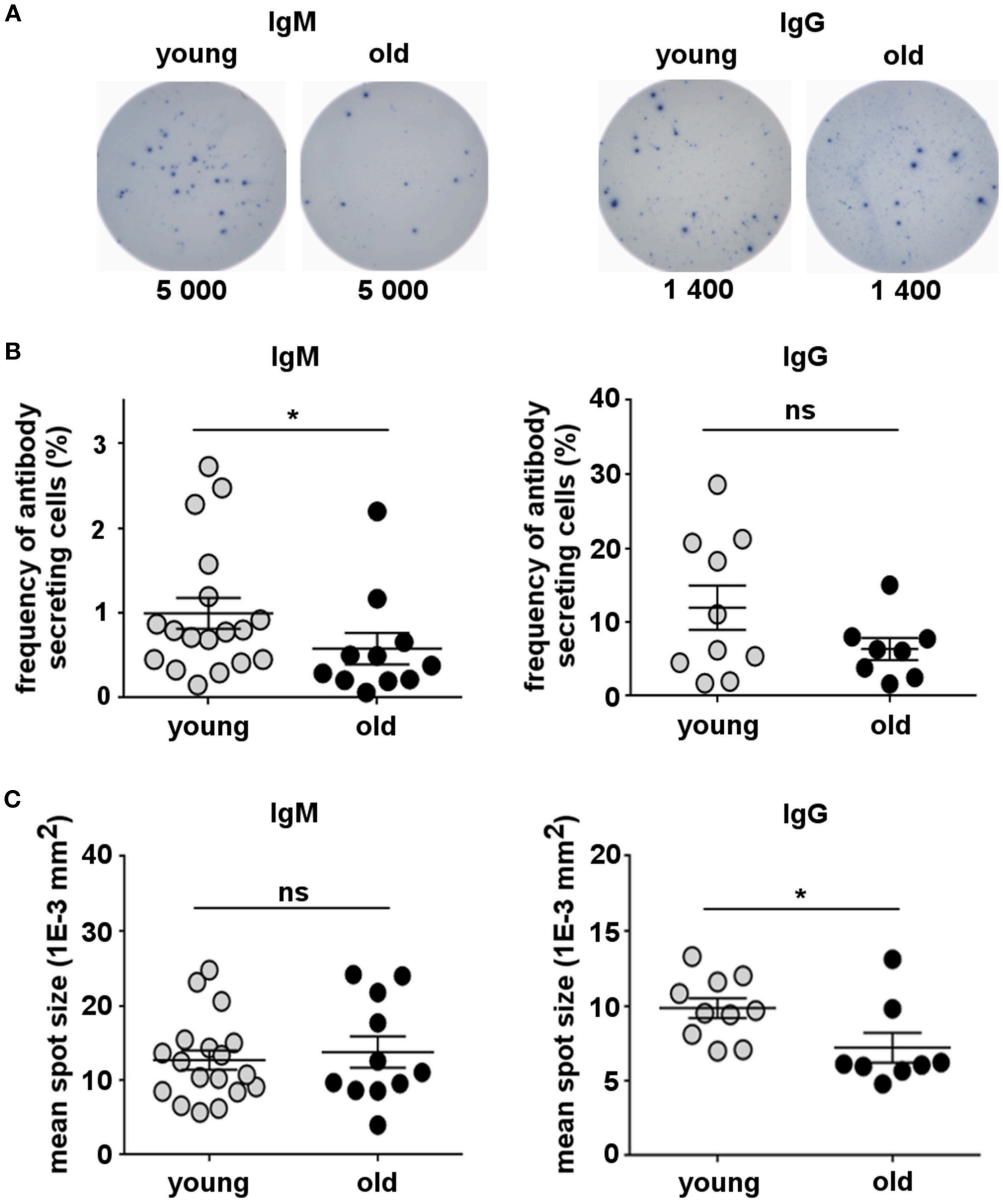
Human B-1 cell spontaneous antibody secretion declines with advancing age. Sort-purified circulating B-1 cells were plated and analyzed for spontaneous IgM and IgG secretion by ELISPOT. **(A)** ELISPOT images corresponding to four different representative donors (2 young and 2 old) are shown. Each sample was tested in duplicate. The number of cells that were plated in each well is indicated. **(B)** Plots show the frequency of IgM- or IgG-secreting B-1 cells from young (*n* = 10–18) and old (*n* = 8–11) donors measured and analyzed by ImmunoSpot software. **(C)** Plots show the mean spot size of IgM- or IgG-secreted antibodies for each young (*n* = 9–18) or old (*n* = 8–10) donor. ^*^p < 0.05 by Mann–Whitney *U*-test, bars are standard errors of the means (SEM).

One of the most important functions of B-lymphocytes is their ability to secrete antibodies. B-1 cells spontaneously secrete IgM and IgG antibodies, which are essential to fight infection (10, 43, 53), and have been related to the pathology of autoimmunity and other diseases (54, 55). Therefore, we investigated whether the ability of B-1 cells to spontaneously secrete antibodies changes with increasing age. Circulating B-1 cells from young (20–45 years) and old (65–88 years) donors were sort-purified and examined for spontaneous IgM and IgG secretion by ELISPOT assay. B-1 cells from both young and old donors spontaneously secreted IgM and IgG antibodies (Figures 2A,B). However, the percentage of B-1 cells spontaneously secreting IgM significantly decreased in old age compared with the young group while the frequency of IgG-secreting B-1 cells from older donors was no different from that of younger donors (Figure 2B). We also compared the amount of IgM or IgG secreted by each individual B-1 cell by calculating mean spot size. B-1 cells from young and old donors secreted similar amounts of IgM per secreting cell (Figure 2C). In contrast, on average, B-1 cells from older donors spontaneously secreted less IgG per secreting cell, compared with B-1 cells from younger individuals (Figure 2C). Thus, the age-related loss of B-1 cells (Figure 1) includes disproportionate loss of IgM-secreting B-1 cells and impaired secretion by an unchanging number of IgG producing cells (Figure 2).

### Human B-1 Cell Expression of Secretory Genes Changes With Advancing Age

**Figure 3:**
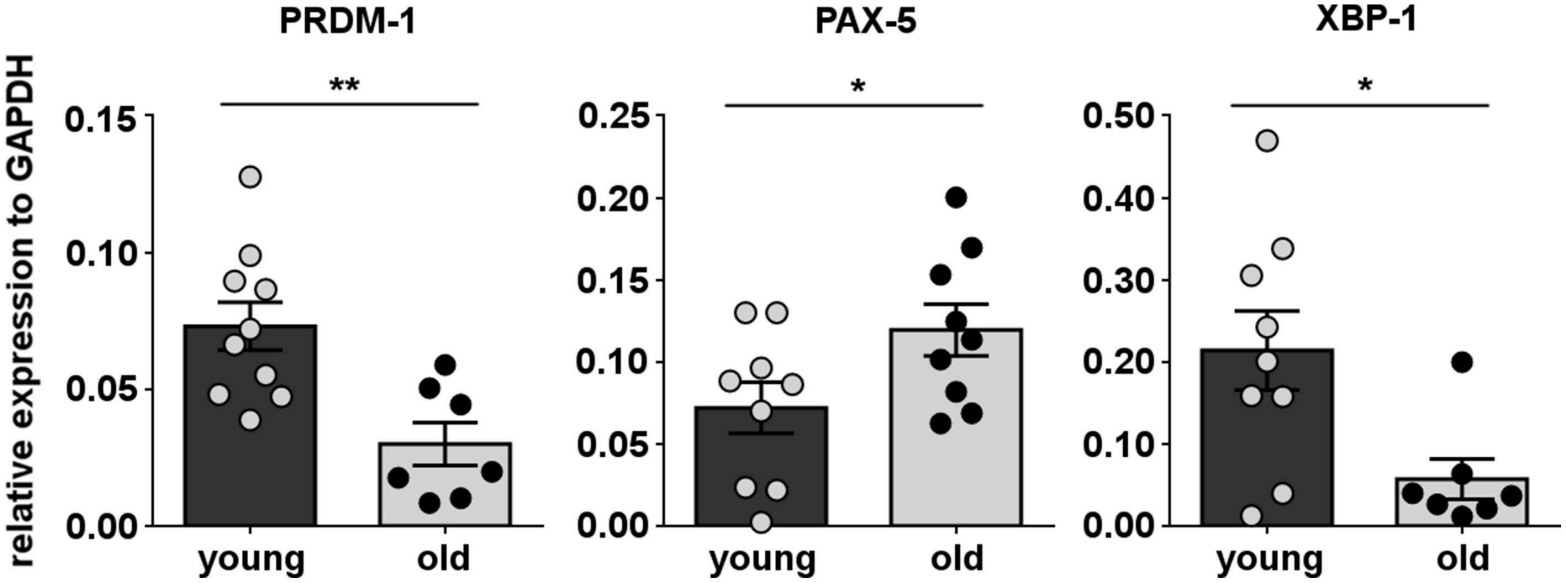
Human B-1 cell expression of secretory genes changes with advancing age. B-1 cells from PBMCs were sort-purified and analyzed by RT-PCR for gene expression. The panels represent the relative expression of transcription factors related with B-cell secretory phenotype for 9–10 young and 7–8 old donors, normalized to GAPDH. ^*^p < 0.05, ^**^p < 0.01 by Mann–Whitney *U*-test, bars are mean and standard error of the mean (SEM).

Immunoglobulin secretion by conventional B-2 cells is controlled by a set of transcription factors including B lymphocyte inducer of maturation program 1 (Blimp-1) encoded by the *PRDM-1* gene locus, Xbox binding protein-1 (XBP-1), and paired box gene 5 (PAX-5) (56). The mechanism of spontaneous immunoglobulin secretion by B-1 cells is only partially understood. However, some of these transcription factors have been shown to be involved in both mouse and human B-1 cell secretion (57, 58). To gain insight into the mechanism underlying downregulation of B-1 cell antibody secretion in aging, we determined whether B-1 cells from old donors exhibit alteration in expression of genes associated with antibody secretion by quantitative RT-PCR, as compared to B-1 cells from young donors. We found the expression levels of *Blimp-1 (PRDM-1)* and *XBP-1*, that promote immunoglobulin secretion, were significantly lower in the B-1 cell population isolated from old donors in contrast with B-1 cells from young donors (Figure 3). Further, with advancing age B-1 cells expressed increased levels of *PAX-5*, an inhibitor of the immunoglobulin secretory phenotype (Figure 3). Thus, the age-related impairment in B-1 cell immunoglobulin secretion could be related to age-related changes in expression of genes that regulate antibody secretion.

### Human B-1 Cell Morphology Changes With Advancing Age

**Figure 4:**
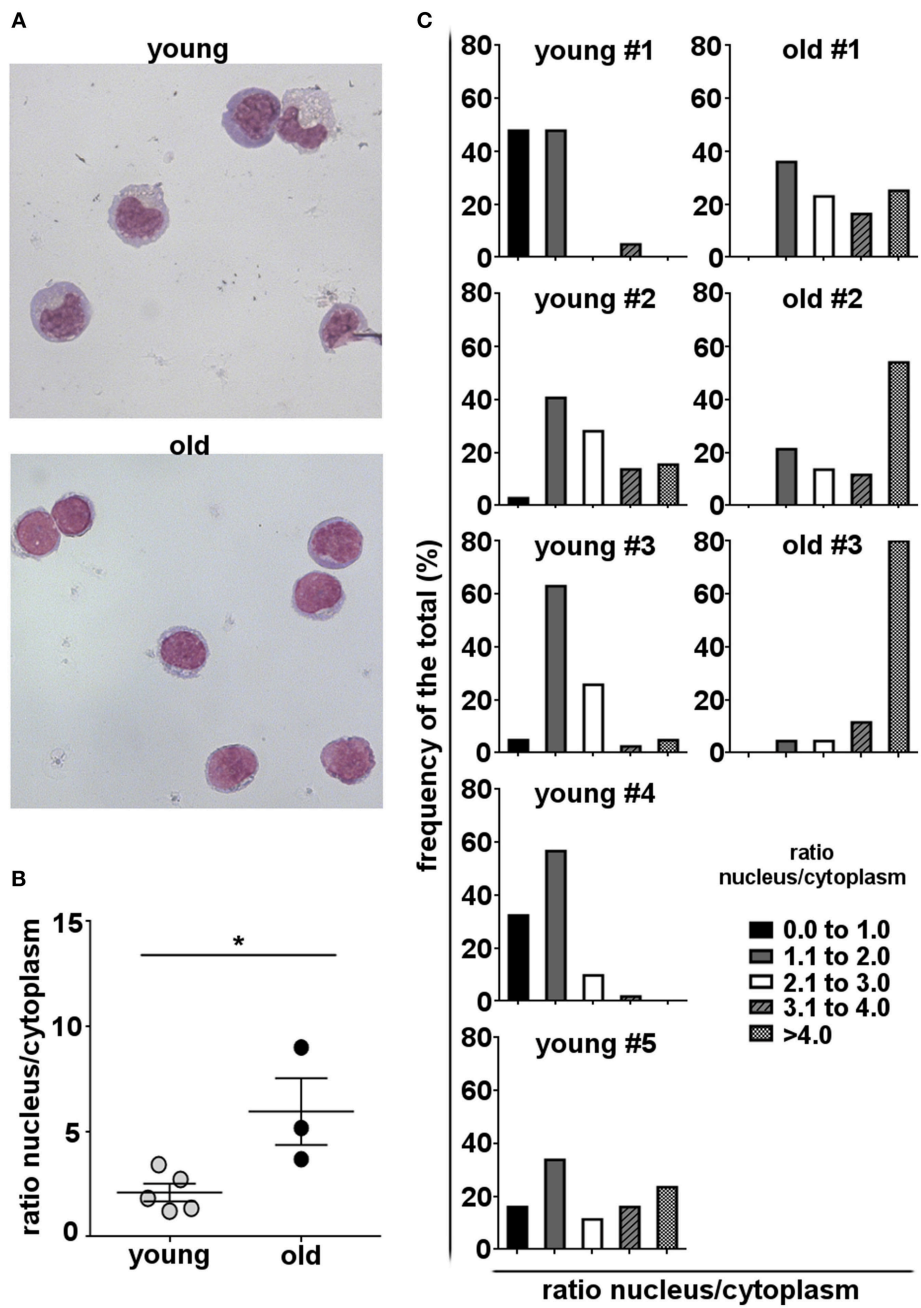
Human B-1 cell morphology changes with advancing age. B-1 cells were sort-purified from PBMCs and stained with Wright–Giemsa stain. **(A)** Representative photomicrographs of B-1 cells from a young and an old donor are shown (magnification 60X). **(B)** Median nucleus/cytoplasm ratios of B-1 cells from young and old donors are shown. The diameters of the nucleus and the cytoplasm were measured by ImageJ software. Each dot represents the median of the nucleus/cytoplasm ratios calculated for each individual donor from the cells in 5–10 fields. ^*^p < 0.05 by Mann–Whitney *U*-test, bars are means and standard errors of the means (SEM). **(C)** Frequency distributions of nucleus/cytoplasm ratios in B-1 cells from five young and 3 old donors are shown.

Antibody secreting cells or plasma cells are characterized by a small, dense eccentrically placed nucleus and prominent cytoplasm, with abundant rough endoplasmic reticulum (RER) and enlarged Golgi. In contrast, non-secreting mature B cells have a high nucleus to cytoplasm ratio, little RER, and an uncondensed nucleus (59–61). To determine whether age-related changes in B-1 cell antibody secretion are reflected in morphological alterations, we examined the appearance of B-1 cells obtained from healthy young and old donors. We found that B-1 cells from young donors displayed morphologic characteristics typical of antibody-secreting cells, such as abundant cytoplasm and a small nucleus. On the other hand, the morphology of B-1 cells from old donors is closer to that of non-secreting B cells with a high nucleus to cytoplasm ratio (Figures 4A,B). Further, the frequency of B-1 cells with a lower nucleus to cytoplasm ratio is higher in each young donor as compared to each old donor (Figure 4C). These results are consistent with results obtained from ELISPOT and gene expression assays, and all together indicate cell-intrinsic defects in the immunoglobulin secretory pathway of B-1 cells accumulate with advancing age.

### Effect of Age on the IgM IGHV Gene Repertoire of Circulating Human B-1 Cells

In humans, the general B cell repertoire of older individuals is characterized by decreased clonal diversity, reduced levels of mutation, and persistent clonal expansions (32, 62, 63). Although the B-1 cell repertoire in particular has been shown to significantly change with age in mice, little is known about equivalent changes in humans. Considering this, we studied age-related changes in the IgM B-1 cell repertoire in healthy humans of young and old age.

**Figure 5:**
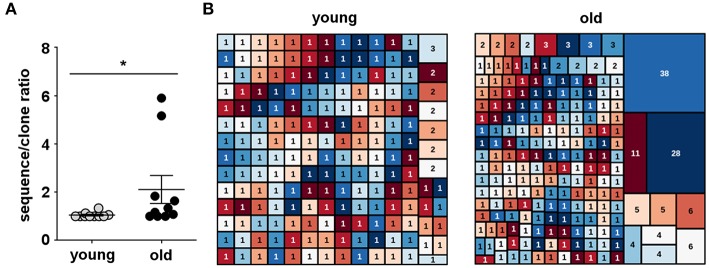
Human B-1 cell antibody diversity declines with advancing age. Single B-1 cells were sorted into 96-well plates, and IgM heavy chains (IGHM) were individually amplified for sequence analysis. IGHM genes were analyzed by IMGT V-QUEST and then clustered into clones by CDR-H3 DNA sequence similarity. **(A)** The total number of IGHM sequences of each donor sample divided by the number of unique IGHM rearrangements in the same sample (sequence/clone ratio) is shown for B-1 cell antibodies from young and old donors. ^*^p < 0.05 by Mann–Whitney *U*-test, bars are means and standard errors of the means (SEM). **(B)** Tree map representation of IGHM repertoires from B-1 cells isolated from young (20–45 years) and old (65–88 years) donors, where each rectangle represents a unique V(D)J rearrangement and the area of each rectangle denotes the relative frequency of an individual sequence. Each graph represents the frequency of IGHM clonotypes in the B-1 cell pool from all the young (*n* = 10) or all the old (*n* = 10) donors. The numbers shown inside the rectangles indicate the sizes of the clones.

We single cell sorted circulating B-1 cells and amplified IgM heavy chain (IGHM) genes. A total of 572 sequences were generated from ten young (20–45 years) and ten old healthy donors (65–88 years). After quality control to ensure only productive, full-length sequences were represented, we obtained a total of 508 IgM sequences: 193 sequences for the young group and 315 sequences for the old group. Overall, a total of 384 unique V(D)J gene rearrangements were identified after heavy chain complementarity determining region 3 (CDR-H3) clustering. Of the 193 sequences obtained from young B-1 cells, 186 were unique sequences (93.6%), while of the 315 sequences obtained from old B-1 cells, 198 were unique sequences (62.8%) (Table 1). The median B-1 cell sequence/clone ratio among young donors was 1.05, with individual values ranging from 1.0 to 1.13 (Figure 5A). In the old donors, however, the sequence/clone ratio was significantly higher, with a median value of 2.11 with individual ratios ranging between 1.0 and 5.90 (Figure 5A). Considering individual donors, only 2 out of 10 B-1 cell samples from young donors had at least one expanded clonotype whereas 7 out of 10 B-1 cell samples from old donors had at least one expanded clonotype.

**Table 1:** Numbers of IGHM sequences produced by single-cell PCR.

	**Young (10 donors)**	**Old (10 donors)**
Total IGHM sequences^*^	193	315
Unique IGHM rearrangements^#^^s^	186	198
Sequence/clone ratio^†^	1.04	1.59

* *The numbers represent the total of IGHM sequences that passed quality control with full immunoglobulin V(D)J gene rearrangement*.

# *The numbers refer to unique sequences, where only one example per clone is counted. Two sequences were considered part of a clonotype if there was a 100% homology in the CDR-H3*.

† *Represents the total number of IGHM sequences of all the young or old donors divided by the number of unique IGHM rearrangements*.

Among young donors, we found 3 clones shared between different individuals while in old donors we did not find any clonotype sharing. Further, four clones were shared between young and old donors. Three out of these four clones were part of expanded clonotypes in either young or old B-1 cells (Table 2). The IgH CDR-H3 tree maps show that B-1 cells from young donors had a highly diverse repertoire (Figure 5B). In contrast, CDR-H3 sequences expressed by B-1 cells from old donors were less diverse and recurred more frequently (Figure 5B). The recurrent CDR-H3 aminoacid sequences for both age groups are summarized in Table 2. Taken together, these data indicate that the B-1 cell IgHM repertoire changes with age and becomes more restricted and repetitive as compared to younger individuals, whose repertoire is more diverse.

**Table 2:** Recurring CDR-H3 sequences (peptide and V(D)J recombination) in B-1 cells from young and old donors.

			**IGHM CDR3 sequences**	
		**Occurrence^**†**^**	**Peptide**	**V(D)J**
YOUNG (20–45 YEARS) (*n =* 10)	1	2	ARAQGSSVDY	V3-21 D2-15 J4
	2	2	ARALGYYDSSGYDLPRIDY^*^	V3-13 D3-22 J4
	3	2	ARDPFADYLDY^$^^*^	V3-74 D3-10 J4
	4	2	ARDWDSSSRDYYGMDV^*^	V3-21 D6-13 J6
	5	2	ARGDPTLSSSSPLDY	V4-34 D6-6 J4
	6	3	AKDIGYTSSSGFDY^$^^*^	V3-9 D6-6 J4
OLD (65–88 YEARS) (*n =* 10)	1	2	AKDEGGGSGWPHYYYQYMDV	V3-9 D6-19 J6
	2	2	ARDQGSGSLHY	V1-3 D3-10 J4
	3	2	AREWLSNSYYYGMDV	V3-21 D3-22 J6
	4	2	AREYSSGWHFDY	V1-3 D6-19 J4
	5	2	ARGGGNWGYYFDS	V4-34 D4-23 J4
	6	2	ARGNYYGSGSYYFDS	V3-7 D3-10 J4
	7	2	ARKIVGATGDDGMDA	V4-34 D1-26 J6
	8	2	VRDAGTGYLDY	V3-30 D5-24 J4
	9	3	ARGGGSPGYSYEFDY	V4-34 D5-18 J4
	10	3	ARHSGWYPYYYMDV	V4-38 D3-10 J6
	11	3	ARSSRYGDYLDAFDI	V3-53 D4-17 J3
	12	3	ARWDCSSTSCVDY^**#**^	V3-30 D2-2 J4
				V3-15 D6-13 J2
	13	4	AKAAYGDYVSTYFDY	V3-30 D4-17 J4
	14	4	AREAFYCTNGLCYLNF	V1-46 D2-8 J4
	15	4	VRDGWGATSNNWFDP^**#**^	V3-7 D1-26 J5
				V3-15 D6-13 J2
	16	5	AMVGYYDTSGQSLDY	V1-18 D3-22 J4
	17	5	TTDLPVADYFQY	V3-15 D6-19 J1
	18	6	ARGDCRGGGCQSHFDS^***$***^	V3-33 D2-15 J4
	19	6	GTDSGAFDM	V3-73 D3-10 J3
	20	11	ARDAIIQYDC	V3-7 D2-8 J4
	21	28	TTVGSGSWFSWYFDL^**#**^	V3-15 D6-13 J2
				V1-8 D6-13 J2
				V3-7 D1-26 J5
				V3-21 D3-22 J4
				V3-30 D3-3 J6
				V4-38 D5-18 J4
	22	38	AGRGLYYDFWSGSTTDYYYMDV	V4-34 D3-9 J6

† *Times a CDR-H3 aminoacid sequence is present in the repertoire of all young or old donors*.

# *The same CDR-H3 aminoacid sequence was encoded by different V(D)J recombination*.

$ *Clonotype sharing between B-1 cell repertoire from young and old donors*.

* Clonotype sharing between B-1 cell repertoire from more than one young (^*^).

**Figure 6:**
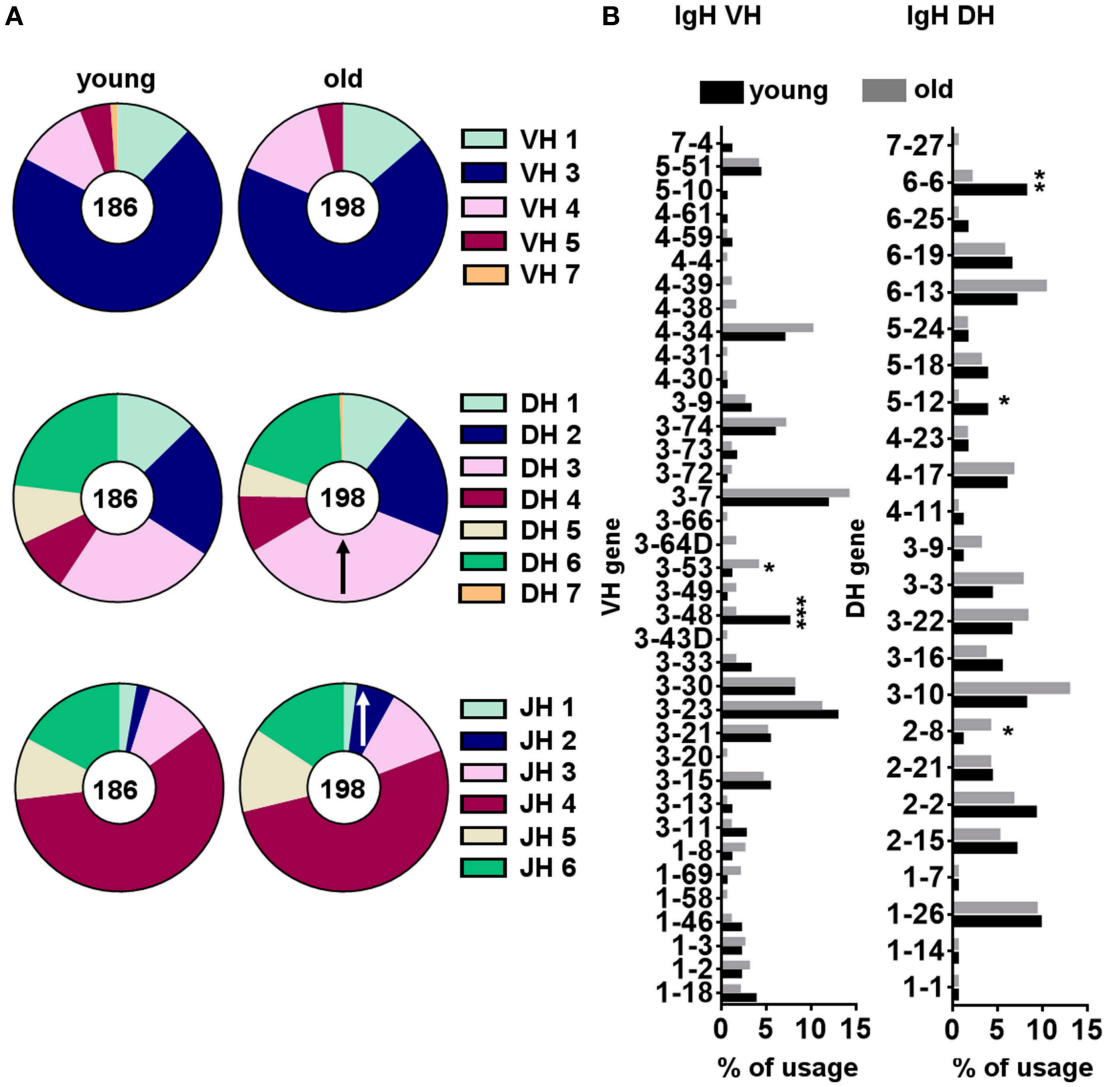
VH, DH and JH gene segment usage frequencies within B-1 cells from young and old donors. Gene usage was calculated as the percentage of the total unique sequences for all the clones from young (*n* = 10) and old (*n* = 10) donors. From each clonotype only one representative sequence was considered for analysis. Relative frequencies of IGHV, IHGD, and IGHJ gene family **(A)**, and individual IGHV and IGHD gene segment **(B)** usage are represented. **↑**indicates a significant increase in IGHD or IGHJ gene family usage in the old B-1 population compared to the corresponding frequency in young B-1 cells. ^*^p < 0.05, ^**^p < 0.01, ^***^p < 0.001 by χ^2^ test or exact Fisher's test.

We quantified the frequency of IGHM sequences expressing individual V_H_, D_H_, and J_H_ genes for each sorted B-1 cell sample and then compared the usage of these segments between young and old donors. From this point onward, only one representative sequence per clone was used for analysis to reduce the influence of clonal expansion on the V_H_, D_H_, and J_H_ gene usage profile. We found B-1 cells from both young and old donors were similar in expressing mainly genes from the IGHV3 family as previously described for this B cell population (20). We also found similarity in use of the IGHD2, IGHD3, and IGHD6 families and the IGHJ4 gene family by B-1 cells from both young and old donors (Figure 6A). However, B-1 cell IgM sequences from old donors differed from those of young donors, at the gene family level, in increased use of IgHD3 and IgHJ2.

Further analysis of the young and old B-1 cell repertoires focused on individual gene segments (Figure 6B). An analysis of individual IGHV and IGHD genes showed a significant increase in IGHV3-53 and IGHD2-8 usage and a significant decrease in IGHV3-48, IGHD5-12, and IGHD6-6 usage with increasing age (Figure 6B). Thus, we identified some changes in IGHM gene segment usage associated with aging, both at the family and the individual gene level, although the significance of these changes within the context of the overall aging repertoire in terms of binding and function are at present unknown.

**Figure 7:**
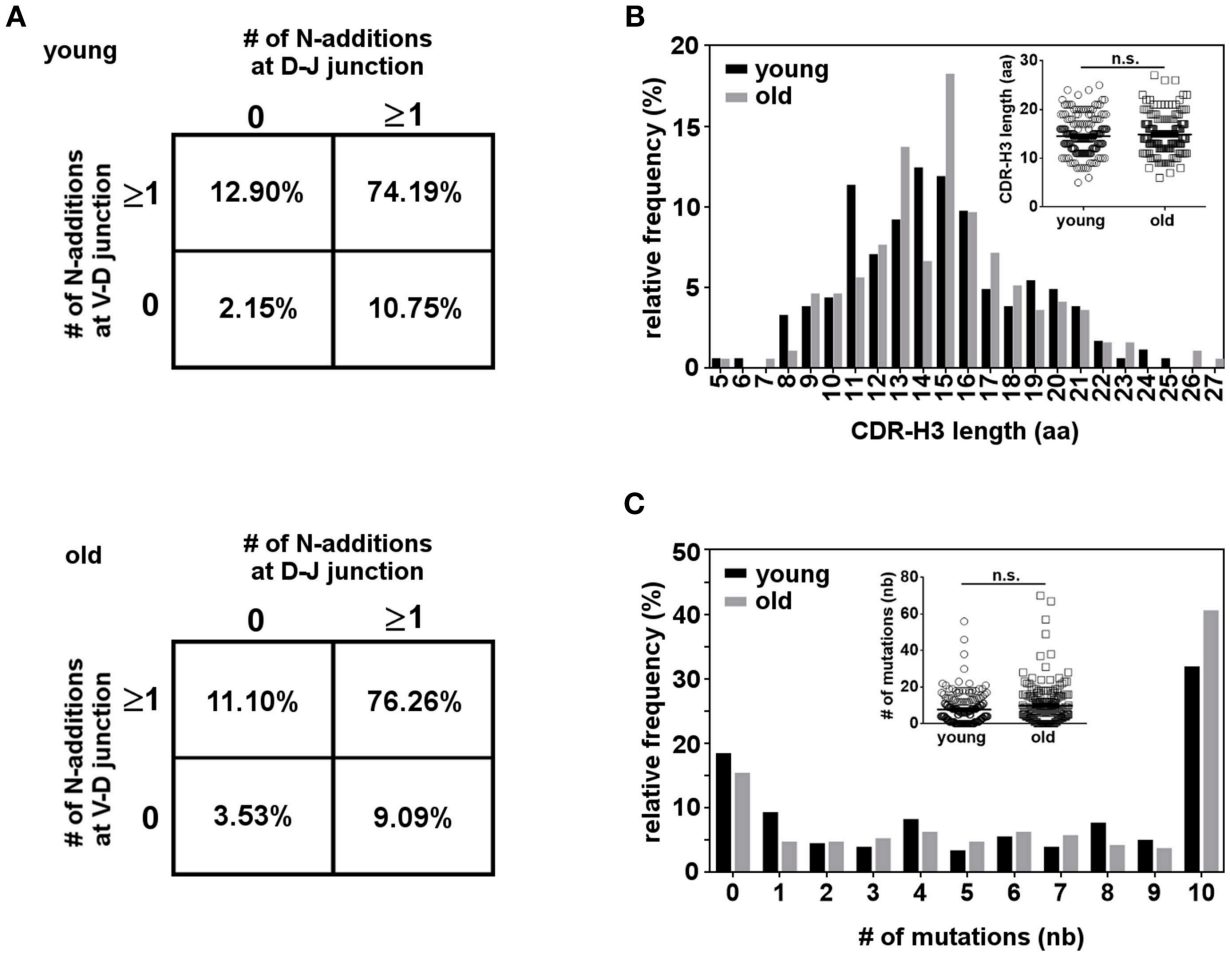
Characterization of the CDR-H3 region in the B-1 cell IGHM repertoire. B-1 cells from 10 young and 10 old donors were single cell sorted and the IgM VH regions were amplified and sequenced. From each clonotype only one representative sequence was considered for analysis. **(A)** N-region addition analysis of B-1 cell IgM from young and old donors. The percentages of sequences with zero N-additions at both junctions, one or more N-additions at both junctions, zero N-additions at V-D and one or more at D-J junctions, or zero N-additions at D-J and one or more at V-D junctions are shown. **(B)** Length distribution of complementarity determining region 3 (CDR-H3) is shown for B-1 cell IgM antibodies from young and old donors. Inset shows the distribution of individual CDR-H3 length from young and old donors **(C)** The distribution of somatic mutations for B-1 cell IgM antibodies from young and old donors is shown as percent of sequences with zero or more than one nucleotide mutation. Inset shows the distribution of mutations for each sequence from young and old donors. n.s. *p* > 0.05 by Mann–Whitney *U*-test.

The CDR-H3 of immunoglobulin molecules is critical for specificity and affinity (64), so alterations in CDR-H3 properties could reflect selection for certain antigens. We compared CDR-H3 characteristics in IGH sequences from young and old B-1 cells. N-region addition provides junctional diversity to the CDR-H3 region through the addition of random nucleotides. Mouse B-1 cell antibodies have few N-additions early in life due to the lack of TdT enzyme during fetal B cell development, but later in life mouse B-1 cell antibodies contain N-additions (45). Human B cells possess N-additions due to constitutive expression of TdT as soon as 12 weeks after gestation (65) but changes with age in human B-1 cells have not been studied. We analyzed N-addition at the D–J and V–D junctions in B-1 cell antibodies from young and old donors. We found only a few antibody sequences did not have any N-addition at either junction (2–4%) and most of the sequences presented more than one N-addition at both junctions (>74%) (Figure 7A). No significant differences were found in the number of N-additions between young and old B-1 cell antibody sequences. We further examined CDR-H3 length and found old B-1 cell antibodies had more CDR-H3 with 13 and 15 aminoacids while young B-1 cell antibodies presented more CDR-H3 with 11 and 14 aminoacids (Figure 7B). However, the average CDR-H3 length did not differ significantly between young and old B-1 cell antibodies (*p* = 0.3570).

B-1 cell IgM antibodies have been shown to have SHM both in mice and humans. Further, the Herzenberg group has found that in mice both splenic and peritoneal B-1 cells accumulate SHM in the IgM repertoire as the animal age (35). Taking this into consideration, we hypothesized that the same might be happening in human B-1 cells. We analyzed somatic hypermutation in B-1 cell antibodies from young and old donors. We found a high percentage of B-1 cell antibody sequences from both young and old donors displayed more than one nucleotide mutation. B-1 cell antibodies without any mutation appear to be less frequent in older as compared to younger donors; further, B-1 cell antibodies with 10 or more mutations tend to be increased in the old group, but this difference did not reach the level of significance (Figure 7C). All together, these results suggest the structure of B-1 cell derived IgM does not change with age, although IgM repertoire diversity decreases significantly.

## Discussion

Previous studies have shown that aging is characterized by significant changes in total B cells and some B cell subpopulations, resulting in age-related changes in antibody composition. However, little is known regarding age-related changes in human B-1 cells. The human B-1 cell phenotype has evolved over time. Early studies suggested that similar to mice, human B-1 cells express CD5 and are present at a higher frequency in human umbilical cord blood and fetal spleen than in adult peripheral blood and spleen (66). Further, human CD5-expressing B cells were reported to produce autoreactive IgM antibodies and to be increased in patients with autoimmune diseases (67, 68). However, CD5 is also present at a variable extent on different human B-cell populations, including transitional, pre-naïve, and activated B-cells (69–71). Further, not all B-1 cells in mice express CD5, as is the case of B-1b cells, suggesting that CD5+ alone is not sufficient to designate the human B-1 cell population. More recently, Griffin et al. proposed a novel phenotype for B-1 cells (CD20+CD27+CD43+) based on the fact that the population so defined fulfills key functional criteria for B-1 cells such as spontaneous antibody secretion (19). Since then, several independent groups have evaluated this circulating B cell population in healthy volunteers, during human fetal hematopoietic development, and in clinical conditions of common variable immunodeficiency, multiple sclerosis, bone marrow transplantation, and lupus (21, 22, 72–74). Here, we identified human B-1 cells as CD19+CD20+CD27+CD38^low/int^ CD43+, according to the more detailed phenotypic profile recently described by Quach et al. (20). In the present study, we found similar percentages of circulating B-1 cells to the ones reported before for this population (20, 22, 23). Although this is a small percentage, similar B-1 frequency has been detected for mouse splenic B-1 cells (75), which are responsible for most of the spontaneous IgM secretion in mice. In fact, we found that the circulating B-1 cells in humans are able to spontaneously secrete immunoglobulin and thus are likely to contribute to the natural antibody pool.

Our results show the percentage of B-1 cells within the total CD19+ B cell population decreases with advancing age. The age-related decline we detected in the human B-1 cell population as a fraction of total B cells is even more striking if the age-related decrease in total CD19+ cells is taken into account. This result is opposite to what has been observed in mice where B-1 cell number increases or remains unchanged with age (76). Mouse B-1 cells arise early in life from embryonic and fetal progenitors and are maintained later on by self-renewal and an unknown level of production from adult bone marrow precursors (45, 77–79). These processes appear not to be affected in a quantitative way in aged mice. Although little is known about human B-1 cell ontogeny, a recent report by Quach et al. showed human B-1 cells develop from umbilical cord blood Lin-CD34+CD38^lo^ stem cells after adoptive transfer to immunodeficient (NSG) mice. However, in the same study Lin-CD34+CD38^lo^ stem cells from adult bone marrow were much less effective in giving rise to B-1 cells (80). The frequency of common lymphoid progenitor cells (81) and, in particular, B cell progenitors (82) has been reported to decrease with age, and human hematopoietic stem cells from old donors, xenotransplanted into immunodeficient mice, do not generate lymphoid progeny as efficiently as stem cells from young donors (81). All together, these findings indicate that poor bone marrow production may be responsible, at least in part, for the age-related decline in total B cells. Thus, poor production of B-1 cells by human adult bone marrow progenitors may contribute to the decline in B-1 cell numbers with advancing age, and/or fail to compensate when B-1 cell numbers decline for other reasons.

It has been shown that aging affects not only the frequency but also the function of B cells (51). The main function of B-1 cells is the spontaneous secretion of antibody. Our results show this important function is adversely affected by age. We found the frequency of spontaneously IgM-secreting B-1 cells was reduced among B-1 cells obtained from old donors as compared to young donors. However, age did not affect the IgM secretion capacity of each individual B-1 cell, which was not impaired. Conversely, the frequency of spontaneously IgG-secreting B-1 cells didn't change as a function of age, but an age-related defect of each spontaneously IgG-secreting B-1 cell was apparent, resulting in less immunoglobulin production per cell. These results might suggest that the mechanism for spontaneous secretion of IgM and IgG by human B-1 cells is different and is affected differently by the aging process.

The mechanism underlying spontaneous secretion of natural antibodies by B-1 cells is not fully understood. Induction of Blimp-1 is one of the first steps required for the generation of antibody secreting plasma cells in mice and in humans (83–85). It has been reported that the mouse B-1 cell subpopulation spontaneously secretes antibodies by mechanisms that are both dependent on, and independent of, Blimp-1, and that work differently for IgM and IgG secretion. Savage et al. demonstrated that splenic B-1 cells spontaneously secreting natural antibodies are a heterogeneous population that contains both Blimp-1-positive B-1 cells and Blimp-1-negative B-1 cells. While both subsets secrete natural IgM, spontaneous IgG3 secretion by B-1 cells was mostly Blimp-1–independent, suggesting an alternative activation/differentiation pathway is used by Blimp-1-negative antibody-secreting cells (58). The mechanism of spontaneous immunoglobulin secretion by human B-1 cells has not been studied; thus, it is unknown whether mice and humans share similar pathways, nor whether human B-1 represent a heterogeneous population regarding the dependency on Blimp-1 for spontaneous secretion. A previous paper by Quach et al. reported that human B-1 cells express substantial levels of Blimp-1 (20), suggesting the importance of this molecule for spontaneous secretion by human B-1 cells. Our results showed that the level of Blimp-1 expression is lower in old B-1 cells as compared to young B-1 cells. Thus, the level of Blimp-1 could be related to a decrease in the frequency of B-1 cells that spontaneously secrete IgM and/or to the fact that old B-1 cells secrete less IgG antibody per cell. The difference we found in the behavior of IgM or IgG secreting B-1 cells with age may be explained by the existence of different pathways for IgM and IgG spontaneous secretion, as was reported for mice.

Blimp-1 deficiency during aging has been reported previously. Frasca et al. found a decrease in Blimp-1 expression in general CD19+ cells after 5–8 days of stimulation with CpG (86). In contrast, in our study we show a significant decrease of Blimp-1 expression in non-stimulated B-1 cells from old individuals compared with young. Our results may represent a defect in the spontaneous/basal expression of Blimp-1 in old B-1 cells which may explain their decreased capacity to spontaneously secrete immunoglobulin. Expression of 2 additional genes tied to antibody secretion also correlated with age-related diminution of B-1 cell antibody secretion: loss of XBP-1 and gain of PAX-5. As XBP-1 is under PAX-5 control and at the same time PAX-5 is regulated by Blimp-1, the difference in the expression levels of the former transcription factors in B-1 cells from old donors may in turn be explained by the observed decrease of Blimp-1 levels. The reason for decreased Blimp-1 mRNA expression in B-1 cells from old as compared to young donors could be due to up-regulation of Blimp-1 repressors (Bach2/MITF) and/or an age-associated down-regulation of the mRNA stability of Blimp-1. In both scenarios, micro-RNAs and other epigenetic modifications may be involved (87), given the fact that epigenetic regulation dramatically changes with age (88, 89) including in the B cell compartment (90, 91). Regardless of the molecular mechanism, with advancing age spontaneous natural antibody secretion by B-1 cells is reduced, in terms of the number of IgM-secreting B-1 cells and the amount of IgG secretion per cell. Further confirmation of age-related alteration in immunoglobulin secretion is provided by morphological changes that include increased nuclear/cytoplasmic ratio in old vs. young B-1 cells.

Next, we further analyzed B-1 IgM antibody repertoire changes during aging. We focused our study on IgM since natural antibodies with this isotype are the ones that have been extensively associated with natural protection against several diseases, many of which are more prevalent in the elderly, and have been associated with homeostatic control in numerous studies in mice and humans (10, 92–96). Beside a cell intrinsic defect in spontaneous immunoglobulin secretion, we found a reduction in IgM antibody heterogeneity among B-1 cells from old donors. There was an increase in the number and size of clones, and a corresponding decrease in the number of unique sequences with advancing age. Previously, Gibson et al. showed that repertoire diversity of total B cells decreases with age and is associated with poor health (32). However, until now there has been no information on the degree to which human B-1 cells participate in this change. In mice, certain splenic B-1a VDJ sequences become progressively more dominant with age (35), and peritoneal B-1a IgM antibody sequences acquire increased levels of N-addition in old animals (43, 45). The origin of these changes in the mouse B-1a repertoire remains unproven but may well result from antigen selection (35, 97, 98). The same may be true of human B-1 cell antibodies. That is, clonal expansion in old individuals may be an adaptive response to recurrent exposure to specific antigens. The decreased diversity of the B-1 cell IgM repertoire in old people might contribute to the weakness of natural antibodies in defense against infections of this population.

Two clones among old B-1 cell antibodies were particularly large (38 and 28 members, respectively). However, because each appeared in only one donor, we consider these subject-specific clones, and it cannot be concluded that these clones represent specificities that are generally increased across the population of older donors. Regardless, it would be of interest to determine the specificities that triggered expansion of these clones. Although definitive information is not available, it is notable that clone 22 (as refered in Table 2) shared 88% identity with an antibody reported in a study about human natural autoantibodies reacting with the α-subunit of the human high-affinity immunoglobulin E (IgE) receptor (FcεRIα) (99).

Discounting clonal expansion, that is, focusing only on unique sequences, there was very little difference in human B-1 cell IgM antibodies from healthy old vs. healthy young donors in terms of VH family usage. This is similar to the situation with human naïve B cells but different than other human B cell subsets (100), and may reflect continual self-renewal of a population established early in life. However, superimposed on this are several distinct differences in antibody usage of individual VH segments between young and old human B-1 cell antibodies, the origin and significance of which remains uncertain.

Contrasting with mouse, in humans, TdT is expressed throughout ontogeny (101). This explains the fact that newborn antibodies from both B-1 and B-2 cells contain N-additions (19, 65). This also might explain why we did not find any increase in the number of N-additions in the old B-1 cell repertoire. Further, the absence of an increased number of N-additions in the elderly corresponds with the similar length of IgM CDR-H3 in young and old B-1 cell antibodies. Although previous reports demonstrated that the old IgM repertoire contains less somatic mutation than the young (63), and that the function of activation-induced cytidine deaminase (AID) is impaired in old B cells as compared to young (51), we did not find any change in the level of SHM in the human B-1 IgM repertoire with age.

Overall our data show that the antibody repertoire of B-1 cells from healthy old individuals differs from that of healthy young individuals in ways that restrict diversity. Together with significant age-related decreases in B-1 cell frequency and IgM/IgG secretion, age-related changes in B-1 cells likely impact the quality of life of the older age population through loss of natural antibody protection.

## Data Availability

The datasets generated for this study can be found in National Center for Biotechnology Information's Genbank, MK433645 - MK434149.

## Author Contributions

NR-Z, TDQ, TLR, and AMH conceived the experiments. NR-Z, TDQ, and TJH carried out the experiments. NR-Z and AMH analyzed data. TLR and AMH oversaw the project. All authors were involved in writing the paper and had final approval of the submitted and published versions.

### Conflict of Interest Statement

The authors declare that the research was conducted in the absence of any commercial or financial relationships that could be construed as a potential conflict of interest.

## Supplementary Material

Supplementary Figure 1 Phenotypic analysis of human peripheral blood B-1 cells by flow cytometry. Lymphocyte-enriched peripheral blood samples were stained with antibodies specific for human CD3, CD4, CD7, CD19, CD20, CD27, CD38, and CD43, and with Aqua Live/Dead dye, and were analyzed by flow cytometry. **(A)** Gating strategy to analyse and purify human B-1 cells is shown. Lymphocytes were subjected to doublet discrimination. Singlet lymphocytes were plotted to exclude CD3, CD4, and CD7 positive cells and to gate on CD19+ B cells. B cells were plotted using CD20 in order to separate CD20+ B cells from CD20- plasma cells. CD20+ B cells were plotted using CD27 and CD38 in order to exclude CD38^high^ pre-plasmablasts. CD38^low/int^ were further resolved according to CD27 and CD43 expression with B-1 cells being those cells expressing both CD27 and CD43. Fluorescence Minus One controls were used for CD43+ and CD27+ cell selection. **(B)** Post-sort analysis and gating strategy for single-cell sorting is shown. For single-cell sorting, purified B-1 cells were re-sorted immediately after the first sorting process according to CD19+CD20+ CD27+CD38^low/int^CD43+ expression applying FSC-H/FSC-W-based doublet discrimination and single sort mask settings.

Supplementary Figure 2 Human B-1 cells decline with advancing age. PBMCs isolated from 87 healthy donors (20–88 years) were analyzed by flow cytometry for total CD19+ B cells **(A)** or B-1 cells (CD19+CD20+CD27+CD38^low/int^CD43+) **(B)**. Distribution of B cells as percent of total lymphocytes **(A)** and B-1 cells as percent CD19+ B cells **(B)** per age range. Different letters represent statistically significant differences; *p* < 0.05, Kruskal-Wallis and Dunn's tests.

## References

[1] Fulop T Dupuis G Witkowski JM Larbi A. The role of immunosenescence in the development of age-related diseases. Rev Invest Clin. (2016) 68:84–91. 27103044

[2] Boraschi D Italiani P. Immunosenescence and vaccine failure in the elderly: strategies for improving response. Immunol Lett. (2014) 162:346–53. 10.1016/j.imlet.2014.06.006 24960535

[3] Del Giudice G Goronzy JJ Grubeck-Loebenstein B Lambert PH Mrkvan T Stoddard JJ. Fighting against a protean enemy: immunosenescence, vaccines, and healthy aging. NPJ Aging Mech Dis. (2018) 4:1. 10.1038/s41514-017-0020-0 29285399 PMC5740164

[4] Scholz JL Diaz A Riley RL Cancro MP Frasca D. A comparative review of aging and B cell function in mice and humans. Curr Opin Immunol. (2013) 25:504–10. 10.1016/j.coi.2013.07.006 23932400 PMC3816359

[5] Hardy RR Hayakawa K. B cell development pathways. Annu Rev Immunol. (2001) 19:595–621. 10.1146/annurev.immunol.19.1.595 11244048

[6] Hardy RR Kincade PW Dorshkind K. The protean nature of cells in the B lymphocyte lineage. Immunity. (2007) 26:703–14. 10.1016/j.immuni.2007.05.013 17582343

[7] De Silva NS Klein U. Dynamics of B cells in germinal centres. Nat Rev Immunol. (2015) 15:137–48. 10.1038/nri3804 25656706 PMC4399774

[8] Baumgarth N Herman OC Jager GC Brown LE Herzenberg LA Chen J. B-1 and B-2 cell-derived immunoglobulin M antibodies are nonredundant components of the protective response to influenza virus infection. J Exp Med. (2000) 192:271–80. 10.1084/jem.192.2.271 10899913 PMC2193249

[9] Alugupalli KR Gerstein RM Chen J Szomolanyi-Tsuda E Woodland RT Leong JM. The resolution of relapsing fever borreliosis requires IgM and is concurrent with expansion of B1b lymphocytes. J Immunol. (2003) 170:3819–27. 10.4049/jimmunol.170.7.3819 12646649

[10] Haas KM Poe JC Steeber DA Tedder TF. B-1a and B-1b cells exhibit distinct developmental requirements and have unique functional roles in innate and adaptive immunity to S. pneumoniae. Immunity. (2005) 23:7–18. 10.1016/j.immuni.2005.04.011 16039575

[11] Baumgarth N. B-1 cell heterogeneity and the regulation of natural and antigen-induced IgM production. Front Immunol. (2016) 7:324. 10.3389/fimmu.2016.00324 27667991 PMC5016532

[12] Tsiantoulas D Diehl CJ Witztum JL Binder CJ. B cells and humoral immunity in atherosclerosis. Circ Res. (2014) 114:1743–56. 10.1161/CIRCRESAHA.113.301145 24855199 PMC4066414

[13] Tornberg UC Holmberg D. B-1a, B-1b and B-2 B cells display unique VHDJH repertoires formed at different stages of ontogeny and under different selection pressures. EMBO J. (1995) 14:1680–9. 10.1002/j.1460-2075.1995.tb07157.x 7737121 PMC398261

[14] Kantor AB Merrill CE Herzenberg LA Hillson JL. An unbiased analysis of V(H)-D-J(H) sequences from B-1a, B-1b, and conventional B cells. J Immunol. (1997) 158:1175–86. 9013957

[15] Gronwall C Vas J Silverman GJ. Protective roles of natural IgM antibodies. Front Immunol. (2012) 3:66. 10.3389/fimmu.2012.00066 22566947 PMC3341951

[16] Kubo T Uchida Y Watanabe Y Abe M Nakamura A Ono M. Augmented TLR9-induced Btk activation in PIR-B-deficient B-1 cells provokes excessive autoantibody production and autoimmunity. J Exp Med. (2009) 206:1971–82. 10.1084/jem.20082392 19687229 PMC2737165

[17] Zhong X Lau S Bai C Degauque N Holodick NE Steven SJ. A novel subpopulation of B-1 cells is enriched with autoreactivity in normal and lupus-prone mice. Arthritis Rheum. (2009) 60:3734–43. 10.1002/art.25015 19950285 PMC2868318

[18] Zhong X Gao W Degauque N Bai C Lu Y Kenny J. Reciprocal generation of Th1/Th17 and T(reg) cells by B1 and B2 B cells. Eur J Immunol. (2007) 37:2400–4. 10.1002/eji.200737296 17683116

[19] Griffin DO Holodick NE Rothstein TL. Human B1 cells in umbilical cord and adult peripheral blood express the novel phenotype CD20+CD27+CD43+CD70. J Exp Med. (2011) 208:67–80. 10.1084/jem.20101499 21220451 PMC3023138

[20] Quach TD Rodriguez-Zhurbenko N Hopkins TJ Guo X Hernandez AM Li W. Distinctions among circulating antibody-secreting cell populations, including B-1 Cells, in human adult peripheral blood. J Immunol. (2016) 196:1060–9. 10.4049/jimmunol.1501843 26740107 PMC5351554

[21] Griffin DO Rothstein TL. A small CD11b(+) human B1 cell subpopulation stimulates T cells and is expanded in lupus. J Exp Med. (2011) 208:2591–8. 10.1084/jem.20110978 22110167 PMC3244038

[22] Torring C Petersen CC Bjerg L Kofod-Olsen E Petersen T Hollsberg P. The B1-cell subpopulation is diminished in patients with relapsing-remitting multiple sclerosis. J Neuroimmunol. (2013) 262:92–9. 10.1016/j.jneuroim.2013.06.002 23856341

[23] Verbinnen B Covens K Moens L Meyts I Bossuyt X. Human CD20+CD43+CD27+CD5- B cells generate antibodies to capsular polysaccharides of *Streptococcus pneumoniae*. J Allergy Clin Immunol. (2012) 130:272–5. 10.1016/j.jaci.2012.04.040 22664161

[24] Birjandi SZ Ippolito JA Ramadorai AK Witte PL. Alterations in marginal zone macrophages and marginal zone B cells in old mice. J Immunol. (2011) 186:3441–51. 10.4049/jimmunol.1001271 21307289 PMC3420341

[25] Hao Y O'Neill P Naradikian MS Scholz JL Cancro MP. A B-cell subset uniquely responsive to innate stimuli accumulates in aged mice. Blood. (2011) 118:1294–304. 10.1182/blood-2011-01-330530 21562046 PMC3152496

[26] Ademokun A Wu YC Dunn-Walters D. The ageing B cell population: composition and function. Biogerontology. (2010) 11:125–37. 10.1007/s10522-009-9256-9 19937382

[27] Frasca D Diaz A Romero M Landin AM Blomberg BB. Age effects on B cells and humoral immunity in humans. Ageing Res Rev. (2011) 10:330–5. 10.1016/j.arr.2010.08.004 20728581 PMC3040253

[28] Sasaki S Sullivan M Narvaez CF Holmes TH Furman D Zheng NY. Limited efficacy of inactivated influenza vaccine in elderly individuals is associated with decreased production of vaccine-specific antibodies. J Clin Invest. (2011) 121:3109–19. 10.1172/JCI57834 21785218 PMC3148747

[29] Frasca D Van der Put E Riley RL Blomberg BB. Reduced Ig class switch in aged mice correlates with decreased E47 and activation-induced cytidine deaminase. J Immunol. (2004) 172:2155–62. 10.4049/jimmunol.172.4.2155 14764681

[30] Riley RL Blomberg BB Frasca D. B cells, E2A, and aging. Immunol Rev. (2005) 205:30–47. 10.1111/j.0105-2896.2005.00268.x 15882343

[31] Miller C Kelsoe G. Ig VH hypermutation is absent in the germinal centers of aged mice. J Immunol. (1995) 155:3377–84. 7561032

[32] Gibson KL Wu YC Barnett Y Duggan O Vaughan R Kondeatis E. B-cell diversity decreases in old age and is correlated with poor health status. Aging Cell. (2009) 8:18–25. 10.1111/j.1474-9726.2008.00443.x 18986373 PMC2667647

[33] Nicoletti C Yang X Cerny J. Repertoire diversity of antibody response to bacterial antigens in aged mice. III Phosphorylcholine antibody from young and aged mice differ in structure and protective activity against infection with *Streptococcus pneumoniae*. J Immunol. (1993) 150:543–9. 8419487

[34] Murasko DM Bernstein ED Gardner EM Gross P Munk G Dran S. Role of humoral and cell-mediated immunity in protection from influenza disease after immunization of healthy elderly. Exp Gerontol. (2002) 37:427–39. 10.1016/S0531-5565(01)00210-8 11772530

[35] Yang Y Wang C Yang Q Kantor AB Chu H Ghosn EE. Distinct mechanisms define murine B cell lineage immunoglobulin heavy chain (IgH) repertoires. Elife. (2015) 4:e09083. 10.7554/eLife.09083 26422511 PMC4714975

[36] Masmoudi H Mota-Santos T Huetz F Coutinho A Cazenave PA. All T15 Id-positive antibodies (but not the majority of VHT15+ antibodies) are produced by peritoneal CD5+ B lymphocytes. Int Immunol. (1990) 2:515–20. 10.1093/intimm/2.6.515 1707658

[37] Wang H Shin DM Abbasi S Jain S Kovalchuk AL Beaty N. Expression of plasma cell alloantigen 1 defines layered development of B-1a B-cell subsets with distinct innate-like functions. Proc Natl Acad Sci USA. (2012) 109:20077–82. 10.1073/pnas.1212428109 23169635 PMC3523855

[38] Adler H Ferreira DM Gordon SB Rylance J. Pneumococcal capsular polysaccharide immunity in the elderly. Clin Vaccine Immunol. (2017) 24:e00004–17. 10.1128/CVI.00004-17 28424198 PMC5461380

[39] Sen G Chen Q Snapper CM. Immunization of aged mice with a pneumococcal conjugate vaccine combined with an unmethylated CpG-containing oligodeoxynucleotide restores defective immunoglobulin G antipolysaccharide responses and specific CD4+-T-cell priming to young adult levels. Infect Immun. (2006) 74:2177–86. 10.1128/IAI.74.4.2177-2186.2006 16552048 PMC1418916

[40] Schenkein JG Park S Nahm MH. Pneumococcal vaccination in older adults induces antibodies with low opsonic capacity and reduced antibody potency. Vaccine. (2008) 26:5521–6. 10.1016/j.vaccine.2008.07.071 18706464 PMC2574975

[41] Lee H Nahm MH Kim KH. The effect of age on the response to the pneumococcal polysaccharide vaccine. BMC Infect Dis. (2010) 10:60. 10.1186/1471-2334-10-60 20219110 PMC2856571

[42] Park S Nahm MH. Older adults have a low capacity to opsonize pneumococci due to low IgM antibody response to pneumococcal vaccinations. Infect Immun. (2011) 79:314–20. 10.1128/IAI.00768-10 21041499 PMC3019891

[43] Holodick NE Vizconde T Hopkins TJ Rothstein TL. Age-related decline in natural igm function: diversification and selection of the B-1a cell pool with age. J Immunol. (2016) 196:4348–57. 10.4049/jimmunol.1600073 27183643 PMC4874569

[44] Gu H Forster I Rajewsky K. Sequence homologies, N sequence insertion and JH gene utilization in VHDJH joining: implications for the joining mechanism and the ontogenetic timing of Ly1 B cell and B-CLL progenitor generation. EMBO J. (1990) 9:2133–40. 10.1002/j.1460-2075.1990.tb07382.x 2113468 PMC551934

[45] Holodick NE Repetny K Zhong X Rothstein TL. Adult BM generates CD5+ B1 cells containing abundant N-region additions. Eur J Immunol. (2009) 39:2383–94. 10.1002/eji.200838920 19714574 PMC2792924

[46] Tiller T Meffre E Yurasov S Tsuiji M Nussenzweig MC Wardemann H. Efficient generation of monoclonal antibodies from single human B cells by single cell RT-PCR and expression vector cloning. J Immunol Methods. (2008) 329:112–24. 10.1016/j.jim.2007.09.017 17996249 PMC2243222

[47] Bulati M Caruso C Colonna-Romano G. From lymphopoiesis to plasma cells differentiation, the age-related modifications of B cell compartment are influenced by “inflamm-ageing.” Ageing Res Rev. (2017) 36:125–36. 10.1016/j.arr.2017.04.001 28396185

[48] Paganelli R Quinti I Fagiolo U Cossarizza A Ortolani C Guerra E. Changes in circulating B cells and immunoglobulin classes and subclasses in a healthy aged population. Clin Exp Immunol. (1992) 90:351–4. 10.1111/j.1365-2249.1992.tb07954.x 1424294 PMC1554614

[49] Shi Y Yamazaki T Okubo Y Uehara Y Sugane K Agematsu K. Regulation of aged humoral immune defense against pneumococcal bacteria by IgM memory B cell. J Immunol. (2005) 175:3262–7. 10.4049/jimmunol.175.5.3262 16116217

[50] Faria AM de Moraes SM de Freitas LH Speziali E Soares TF Figueiredo-Neves SP. Variation rhythms of lymphocyte subsets during healthy aging. Neuroimmunomodulation. (2008) 15:365–79. 10.1159/000156478 19047812

[51] Frasca D Landin AM Lechner SC Ryan JG Schwartz R Riley RL. Aging down-regulates the transcription factor E2A, activation-induced cytidine deaminase, and Ig class switch in human B cells. J Immunol. (2008) 180:5283–90. 10.4049/jimmunol.180.8.5283 18390709

[52] Lin Y Kim J Metter EJ Nguyen H Truong T Lustig A. Changes in blood lymphocyte numbers with age *in vivo* and their association with the levels of cytokines/cytokine receptors. Immun Ageing. (2016) 13:24. 10.1186/s12979-016-0079-7 27547234 PMC4990976

[53] Choi YS Dieter JA Rothaeusler K Luo Z Baumgarth N. B-1 cells in the bone marrow are a significant source of natural IgM. Eur J Immunol. (2012) 42:120–9. 10.1002/eji.201141890 22009734 PMC3426357

[54] Takahashi T Strober S. Natural killer T cells and innate immune B cells from lupus-prone NZB/W mice interact to generate IgM and IgG autoantibodies. Eur J Immunol. (2008) 38:156–65. 10.1002/eji.200737656 18050273 PMC2915938

[55] Enghard P Humrich JY Chu VT Grussie E Hiepe F Burmester GR. Class switching and consecutive loss of dsDNA-reactive B1a B cells from the peritoneal cavity during murine lupus development. Eur J Immunol. (2010) 40:1809–18. 10.1002/eji.200940050 20333624

[56] Nutt SL Hodgkin PD Tarlinton DM Corcoran LM. The generation of antibody-secreting plasma cells. Nat Rev Immunol. (2015) 15:160–71. 10.1038/nri3795 25698678

[57] Inui M Hirota S Hirano K Fujii H Sugahara-Tobinai A Ishii T Human CD43+ B cells are closely related not only to memory B cells phenotypically but also to plasmablasts developmentally in healthy individuals. Int Immunol. (2015) 27:345–55. 10.1093/intimm/dxv009 25744616

[58] Savage HP Yenson VM Sawhney SS Mousseau BJ Lund FE Baumgarth N. Blimp-1-dependent and -independent natural antibody production by B-1 and B-1-derived plasma cells. J Exp Med. (2017) 214:2777–94. 10.1084/jem.20161122 28698287 PMC5584113

[59] Agematsu K Nagumo H Yang FC Nakazawa T Fukushima K Ito S. B cell subpopulations separated by CD27 and crucial collaboration of CD27+ B cells and helper T cells in immunoglobulin production. Eur J Immunol. (1997) 27:2073–9. 10.1002/eji.1830270835 9295047

[60] Fecteau JF Cote G Neron S. A new memory CD27-IgG+ B cell population in peripheral blood expressing VH genes with low frequency of somatic mutation. J Immunol. (2006) 177:3728–36. 10.4049/jimmunol.177.6.3728 16951333

[61] Halliley JL Tipton CM Liesveld J Rosenberg AF Darce J Gregoretti IV. Long-lived plasma cells are contained within the CD19(-)CD38(hi)CD138(+) subset in human bone marrow. Immunity. (2015) 43:132–45. 10.1016/j.immuni.2015.06.016 26187412 PMC4680845

[62] Buffa S Bulati M Pellicano M Dunn-Walters DK Wu YC Candore G. B cell immunosenescence: different features of naive and memory B cells in elderly. Biogerontology. (2011) 12:473–83. 10.1007/s10522-011-9353-4 21879287

[63] Wu YC Kipling D Dunn-Walters DK. Age-related changes in human peripheral blood IGH repertoire following vaccination. Front Immunol. (2012) 3:193. 10.3389/fimmu.2012.00193 22787463 PMC3391689

[64] Kirkham PM Schroeder HW Jr. Antibody structure and the evolution of immunoglobulin V gene segments. Semin Immunol. (1994) 6:347–60. 10.1006/smim.1994.1045 7654992

[65] Rechavi E Lev A Lee YN Simon AJ Yinon Y Lipitz S. Timely and spatially regulated maturation of B and T cell repertoire during human fetal development. Sci Transl Med. (2015) 7:276ra225. 10.1126/scitranslmed.aaa0072 25717098

[66] Antin JH Emerson SG Martin P Gadol N Ault KA. Leu-1+ (CD5+) B cells. A major lymphoid subpopulation in human fetal spleen: phenotypic and functional studies. J Immunol. (1986) 136:505–10. 2934473

[67] Casali P Burastero SE Nakamura M Inghirami G Notkins AL. Human lymphocytes making rheumatoid factor and antibody to ssDNA belong to Leu-1+ B-cell subset. Science. (1987) 236:77–81. 10.1126/science.3105056 3105056

[68] Hardy RR Hayakawa K Shimizu M Yamasaki K Kishimoto T. Rheumatoid factor secretion from human Leu-1+ B cells. Science. (1987) 236:81–3. 10.1126/science.3105057 3105057

[69] Freedman AS Freeman G Whitman J Segil J Daley J Nadler LM. Studies of *in vitro* activated CD5+ B cells. Blood. (1989) 73:202–8. 2462935

[70] Sims GP Ettinger R Shirota Y Yarboro CH Illei GG Lipsky PE. Identification and characterization of circulating human transitional B cells. Blood. (2005) 105:4390–8. 10.1182/blood-2004-11-4284 15701725 PMC1895038

[71] Lee J Kuchen S Fischer R Chang S Lipsky PE. Identification and characterization of a human CD5+ pre-naive B cell population. J Immunol. (2009) 182:4116–26. 10.4049/jimmunol.0803391 19299709

[72] Kraljevic K Wong S Fulcher DA. Circulating phenotypic B-1 cells are decreased in common variable immunodeficiency and correlate with immunoglobulin M levels. Clin Exp Immunol. (2013) 171:278–82. 10.1111/cei.12008 23379434 PMC3569535

[73] Moins-Teisserenc H Busson M Herda A Apete S Peffault de Latour R Robin M. CD19+CD5+ B cells and B1-like cells following allogeneic hematopoietic stem cell transplantation. Biol Blood Marrow Transplant. (2013) 19:988–91. 10.1016/j.bbmt.2013.03.006 23507469

[74] Bueno C van Roon EH Munoz-Lopez A Sanjuan-Pla A Juan M Navarro A. Immunophenotypic analysis and quantification of B-1 and B-2 B cells during human fetal hematopoietic development. Leukemia. (2016) 30:1603–6. 10.1038/leu.2015.362 26710885

[75] Baumgarth N Waffarn EE Nguyen TT. Natural and induced B-1 cell immunity to infections raises questions of nature versus nurture. Ann N Y Acad Sci. (2015) 1362:188–99. 10.1111/nyas.12804 26060895 PMC4881423

[76] Haas KM Blevins MW High KP Pang B Swords WE Yammani RD Aging promotes B-1b cell responses to native, but not protein-conjugated, pneumococcal polysaccharides: implications for vaccine protection in older adults. J Infect Dis. (2014) 209:87–97. 10.1093/infdis/jit442 23964109 PMC3864388

[77] Duber S Hafner M Krey M Lienenklaus S Roy B Hobeika E. Induction of B-cell development in adult mice reveals the ability of bone marrow to produce B-1a cells. Blood. (2009) 114:4960–7. 10.1182/blood-2009-04-218156 19812384

[78] Yoshimoto M Montecino-Rodriguez E Ferkowicz MJ Porayette P Shelley WC Conway SJ. Embryonic day 9 yolk sac and intra-embryonic hemogenic endothelium independently generate a B-1 and marginal zone progenitor lacking B-2 potential. Proc Natl Acad Sci USA. (2011) 108:1468–73. 10.1073/pnas.1015841108 21209332 PMC3029764

[79] Ghosn EE Waters J Phillips M Yamamoto R Long BR Yang Y. Fetal hematopoietic stem cell transplantation fails to fully regenerate the B-lymphocyte compartment. Stem Cell Reports. (2016) 6:137–49. 10.1016/j.stemcr.2015.11.011 26724903 PMC4720028

[80] Quach TD Hopkins TJ Holodick NE Vuyyuru R Manser T Bayer RL. Human B-1 and B-2 B cells develop from Lin-CD34+CD38lo stem cells. J Immunol. (2016) 197:3950–8. 10.4049/jimmunol.1600630 27815443 PMC5363078

[81] Pang WW Price EA Sahoo D Beerman I Maloney WJ Rossi DJ. Human bone marrow hematopoietic stem cells are increased in frequency and myeloid-biased with age. Proc Natl Acad Sci USA. (2011) 108:20012–7. 10.1073/pnas.1116110108 22123971 PMC3250139

[82] Kuranda K Vargaftig J de la Rochere P Dosquet C Charron D Bardin F. Age-related changes in human hematopoietic stem/progenitor cells. Aging Cell. (2011)10:542–6. 10.1111/j.1474-9726.2011.00675.x 21418508

[83] Shapiro-Shelef M Lin KI McHeyzer-Williams LJ Liao J McHeyzer-Williams MG Calame K. Blimp-1 is required for the formation of immunoglobulin secreting plasma cells and pre-plasma memory B cells. Immunity. (2003) 19:607–20. 10.1016/S1074-7613(03)00267-X 14563324

[84] Ding BB Bi E Chen H Yu JJ Ye BH. IL-21 and CD40L synergistically promote plasma cell differentiation through upregulation of Blimp-1 in human B cells. J Immunol. (2013) 190:1827–36. 10.4049/jimmunol.1201678 23325890 PMC3563840

[85] Tellier J Shi W Minnich M Liao Y Crawford S Smyth GK. Blimp-1 controls plasma cell function through the regulation of immunoglobulin secretion and the unfolded protein response. Nat Immunol. (2016) 17:323–30. 10.1038/ni.3348 26779600 PMC4757736

[86] Frasca D Diaz A Romero M Blomberg BB The generation of memory B cells is maintained, but the antibody response is not, in the elderly after repeated influenza immunizations. Vaccine. (2016) 34:2834–40. 10.1016/j.vaccine.2016.04.023 27108193 PMC4876633

[87] Tanaka H Muto A Shima H Katoh Y Sax N Tajima S. Epigenetic regulation of the blimp-1 gene (Prdm1) in B cells involves bach2 and histone deacetylase 3. J Biol Chem. (2016) 291:6316–30. 10.1074/jbc.M116.713842 26786103 PMC4813568

[88] Fraga MF Esteller M. Epigenetics and aging: the targets and the marks. Trends Genet. (2007) 23:413–8. 10.1016/j.tig.2007.05.008 17559965

[89] Gonzalo S. Epigenetic alterations in aging. J Appl Physiol. (2010) 109:586–97. 10.1152/japplphysiol.00238.2010 20448029 PMC2928596

[90] Frasca D Diaz A Romero M Ferracci F Blomberg BB. MicroRNAs miR-155 and miR-16 decrease AID and E47 in B cells from elderly individuals. J Immunol. (2015) 195:2134–40. 10.4049/jimmunol.1500520 26223652 PMC4546853

[91] Wu H Deng Y Feng Y Long D Ma K Wang X. Epigenetic regulation in B-cell maturation and its dysregulation in autoimmunity. Cell Mol Immunol. (2018) 15:676–84. 10.1038/cmi.2017.133 29375128 PMC6123482

[92] Boes M Prodeus AP Schmidt T Carroll MC Chen J. A critical role of natural immunoglobulin M in immediate defense against systemic bacterial infection. J Exp Med. (1998) 188:2381–6. 10.1084/jem.188.12.2381 9858525 PMC2212438

[93] Lewis MJ Malik TH Ehrenstein MR Boyle JJ Botto M Haskard DO. Immunoglobulin M is required for protection against atherosclerosis in low-density lipoprotein receptor-deficient mice. Circulation. (2009) 120:417–26. 10.1161/CIRCULATIONAHA.109.868158 19620499 PMC2761224

[94] Kyaw T Tipping P Bobik A Toh BH. Protective role of natural IgM-producing B1a cells in atherosclerosis. Trends Cardiovasc Med. (2012) 22:48–53. 10.1016/j.tcm.2012.06.011 22841841

[95] Giamarellos-Bourboulis EJ Apostolidou E Lada M Perdios I Gatselis NK Tsangaris I. Kinetics of circulating immunoglobulin M in sepsis: relationship with final outcome. Crit Care. (2013) 17:R247. 10.1186/cc13073 24144038 PMC4056013

[96] Engelbertsen D Vallejo J Quach TD Fredrikson GN Alm R Hedblad B. Low levels of IgM antibodies against an advanced glycation endproduct-modified apolipoprotein B100 peptide predict cardiovascular events in nondiabetic subjects. J Immunol. (2015) 195:3020–5. 10.4049/jimmunol.1402869 26290603

[97] Cole LE Yang Y Elkins KL Fernandez ET Qureshi N Shlomchik MJ. Antigen-specific B-1a antibodies induced by Francisella tularensis LPS provide long-term protection against *F. tularensis LVS challenge*. Proc Natl Acad Sci USA. (2009) 106:4343–8. 10.1073/pnas.0813411106 19251656 PMC2657382

[98] Holodick NE Rodriguez-Zhurbenko N Hernandez AM. Defining natural antibodies. Front Immunol. (2017) 8:872. 10.3389/fimmu.2017.00872 28798747 PMC5526850

[99] Horn MP Pachlopnik JM Vogel M Dahinden M Wurm F Stadler BM. Conditional autoimmunity mediated by human natural anti-Fc(epsilon)RIalpha autoantibodies? FASEB J. (2001) 15:2268–74. 10.1096/fj.00-0890hyp 11641254

[100] Martin V Wu YC Kipling D Dunn-Walters DK. Age-related aspects of human IgM(+) B cell heterogeneity. Ann N Y Acad Sci. (2015) 1362:153–63. 10.1111/nyas.12823 26152370 PMC4758400

[101] Asma GE van den Bergh RL Vossen JM. Characterization of early lymphoid precursor cells in the human fetus using monoclonal antibodies and anti-terminal deoxynucleotidyl transferase. Clin Exp Immunol. (1986) 64:356–63. 3742878 PMC1542329

