# Supplementary figures and images for: Human B-1 Cells and B-1 Cell Antibodies Change With Advancing Age
Source: Front Immunol. 2019 Mar 19;10:483. doi: 10.3389/fimmu.2019.00483 (PMC6433875; doi:10.3389/fimmu.2019.00483)

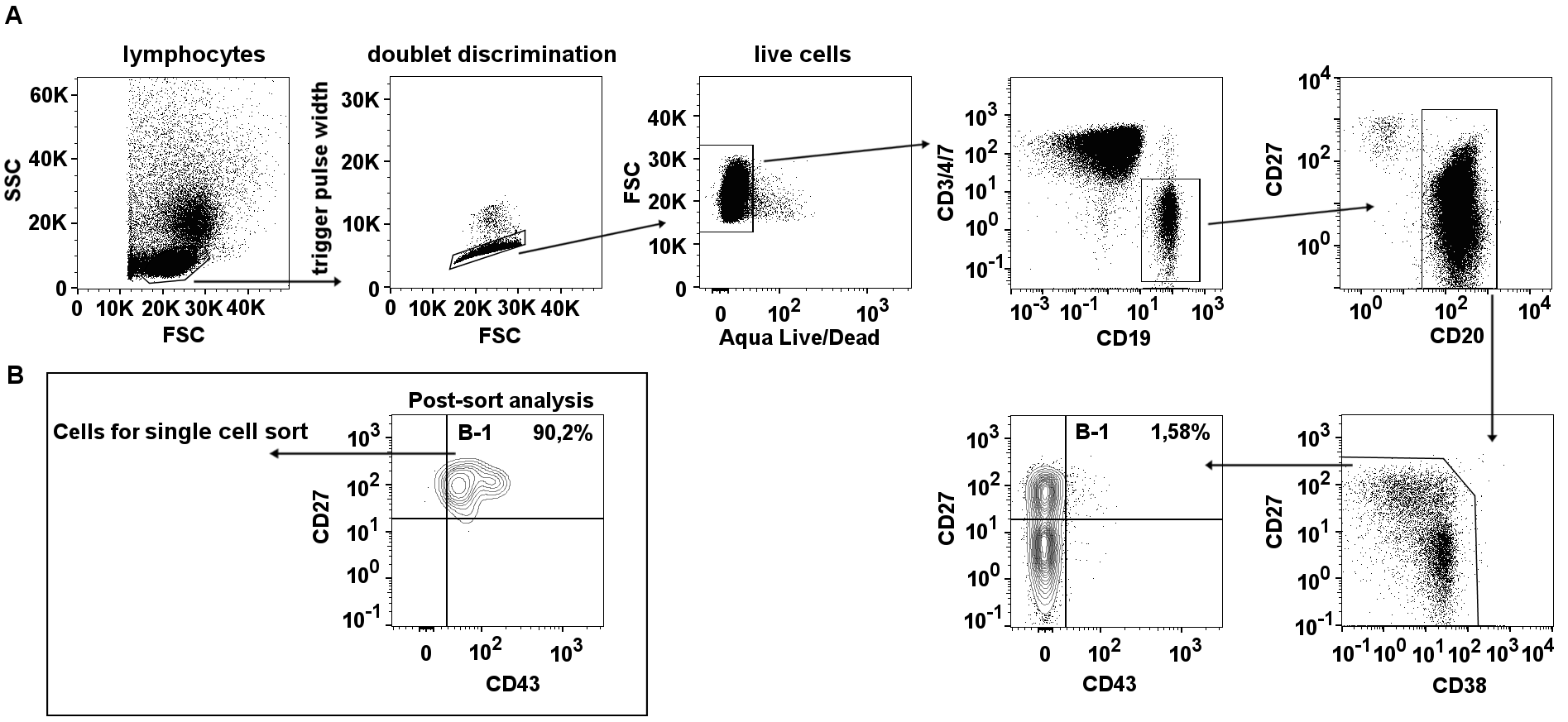

Supplement: Supplementary Figure 1 — Phenotypic analysis of human peripheral blood B-1 cells by flow cytometry. Lymphocyte-enriched peripheral blood samples were stained with antibodies specific for human CD3, CD4, CD7, CD19, CD20, CD27, CD38, and CD43, and with Aqua Live/Dead dye, and were analyzed by flow cytometry. (A) Gating strategy to analyse and purify human B-1 cells is shown. Lymphocytes were subjected to doublet discrimination. Singlet lymphocytes were plotted to exclude CD3, CD4, and CD7 positive cells and to gate on CD19+ B cells. B cells were plotted using CD20 in order to separate CD20+ B cells from CD20- plasma cells. CD20+ B cells were plotted using CD27 and CD38 in order to exclude CD38high pre-plasmablasts. CD38low/int were further resolved according to CD27 and CD43 expression with B-1 cells being those cells expressing both CD27 and CD43. Fluorescence Minus One controls were used for CD43+ and CD27+ cell selection. (B) Post-sort analysis and gating strategy for single-cell sorting is shown. For single-cell sorting, purified B-1 cells were re-sorted immediately after the first sorting process according to CD19+CD20+ CD27+CD38low/intCD43+ expression applying FSC-H/FSC-W-based doublet discrimination and single sort mask settings. [file Image_1.TIF]

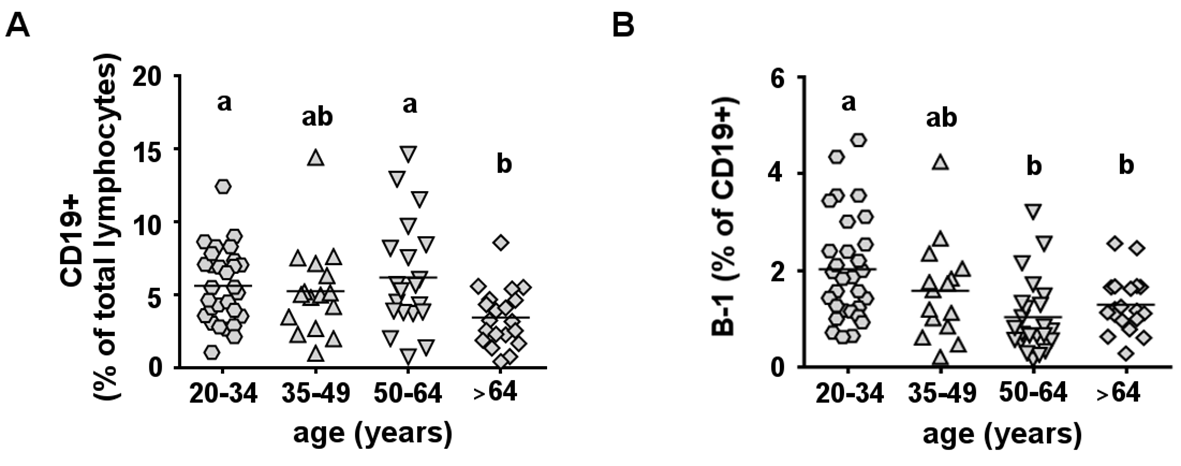

Supplement: Supplementary Figure 2 — Human B-1 cells decline with advancing age. PBMCs isolated from 87 healthy donors (20–88 years) were analyzed by flow cytometry for total CD19+ B cells (A) or B-1 cells (CD19+CD20+CD27+CD38low/intCD43+) (B). Distribution of B cells as percent of total lymphocytes (A) and B-1 cells as percent CD19+ B cells (B) per age range. Different letters represent statistically significant differences; p < 0.05, Kruskal-Wallis and Dunn's tests. [file Image_2.TIF]
